# Representative Scanpath Identification for Group Viewing Pattern Analysis

**DOI:** 10.16910/jemr.11.6.5

**Published:** 2018-11-22

**Authors:** Aoqi Li, Zhenzhong Chen

**Affiliations:** Wuhan University, Wuhan, China

**Keywords:** eye movement, eye tracking, representative scanpath, attention, viewing pattern, barycenter, gaze duration

## Abstract

Scanpaths are composed of fixations and saccades. Viewing trends reflected by scanpaths play an important role in scientific studies like saccadic model evaluation and real-life applications like artistic design. Several scanpath synthesis methods have been proposed to obtain a scanpath that is representative of the group viewing trend. But most of them either target a specific category of viewing materials like webpages or leave out some useful information like gaze duration. Our previous work defined the representative scanpath as the barycenter of a group of scanpaths, which actually shows the averaged shape of multiple scanpaths. In this paper, we extend our previous framework to take gaze duration into account, obtaining representative scanpaths that describe not only attention distribution and shift but also attention span. The extended framework consists of three steps: Eye-gaze data preprocessing, scanpath aggregation and gaze duration analysis. Experiments demonstrate that the framework can well serve the purpose of mining viewing patterns and “barycenter” based representative scanpaths can better characterize the pattern.

## Introduction

Vision is the main channel through which humans acquire external information. Based on the eye-mind hypothesis (1), what subjects see can help to predict human's cognitive activities such as user intentions (2), sarcasm understandability (3), risky decision making (4) and reading effort (5). Eye trackers record human viewing behavior in the form of raw eye tracking data, which can be processed into scanpaths (6) composed of fixations and saccades.

**Figure 1.**
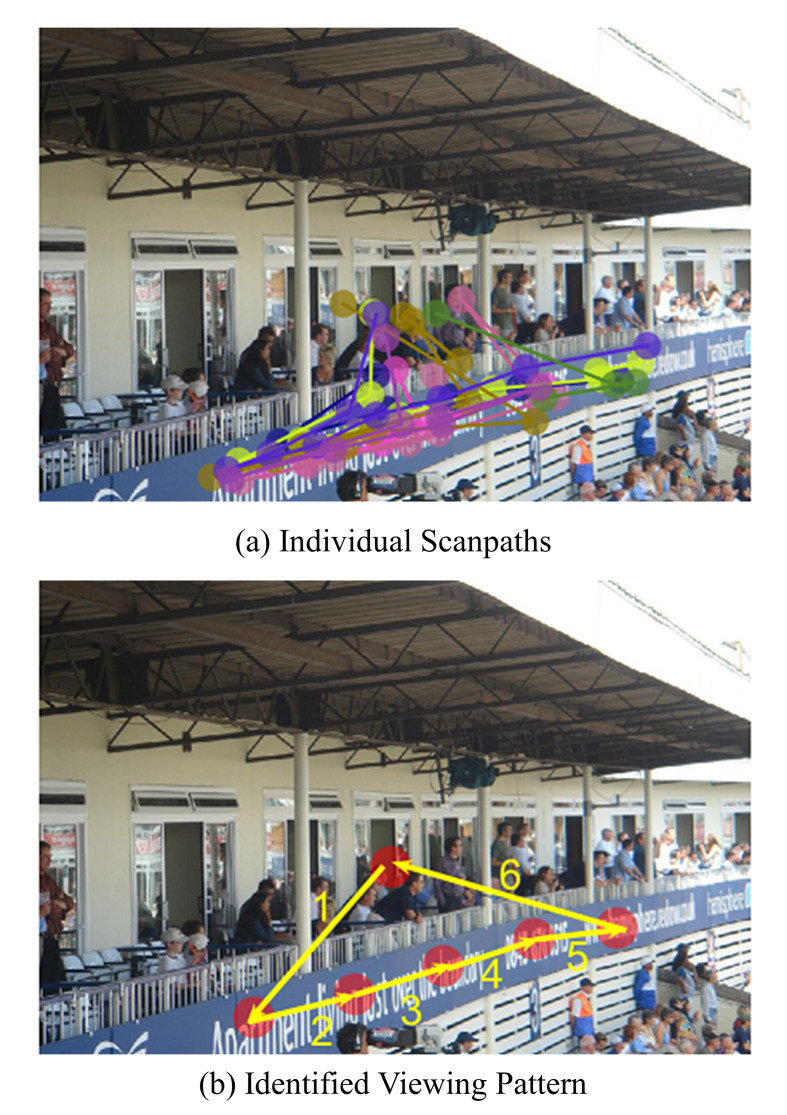
An example to illustrate the viewing pattern for a natural image from MIT1003 dataset.

Scanpaths reflect the ebbs and flows of visual attention. According to Yarbus’ research (7), scanpaths from different observers for the same visual stimuli in free viewing conditions are similar but not identical. The scanning order of one subject is not perfectly congruent with that of others as shown in Figure 1 (a), so it remains a challenging task to identify from multiple scanpaths a pattern that reflects the attention synchrony of different subjects as shown in Figure 1 (b). Such a pattern not only plays an important role in understanding how humans perceive and explore their surrounding scenes but also reveals some important properties of visual stimuli, so it has a wide range of applications in many fields. For example, in psychology, it can be used to identify reading habits of experts and detect reading disorder; in marketing, it can tell us which parts of an advertisement first grab customer attention and help to design a more user-friendly interface; in computer vision, it can be regarded as the group viewing pattern to train a network for scanpath prediction.

## Related Work

Several methods were proposed to analyze scanpaths. For example, T-pattern is a tool to discover repetitive scan patterns in each individual scanpath (8, 9). Others attempt to characterize complex scanning patterns in dynamic tasks such as air traffic control (10). However, to get the group viewing pattern, we need to take into account all individual scanpaths rather than focus on a single one, like the identified scanpath in Figure 1(b), which we call representative scanpath. The surge of interest in dynamic visual attention gives rise to various methods for representative scanpaths identification, most of which either stem from sequence mining algorithms or target a specific category of visual stimuli such as web pages (11, 12, 13, 14, 15, 16). So they have limitations when applied to analyze scanpaths. Existing methods to analyze scanpaths include extracting common subsequences shared by all the subjects (11, 17, 18, 19). However, in the case where there is no common component shared by individual scanpaths, methods in this category will fail to produce any pattern. To be more tolerant of individual differences, sequential pattern mining algorithms can be used to obtain frequent subsequences supported by a specified number of subjects (20). But a fixed threshold of subject number can hardly be suitable for all the images due to the varying degree of scanpath inconsistency incurred by personal viewing habits and visual stimuli properties. Hence produced subsequences may still be too short to reflect the complete viewing pattern. Instead of simply focusing on subsequences, scanpath trend analysis (STA) (13) is proposed to acquire the viewing pattern from a whole new perspective. STA first selects representatively trending instances from scanpath components and then rearranges them based on their average rank in all the individual scanpaths. To make STA more tolerant, a new parameter *tolerance level*, which allows trending instances to be shared by a subset of scanpaths rather than all of them, is added to the original STA algorithm (16), but it is difficult to propose a specific *tolerance level*. The main limitation of STA and its variant is that it targets web pages and relies on the natural segmentation of visual elements (e.g., navigation bar, text box, etc.) to denote scanpaths by character strings. Apart from the above studies, researchers in computer vision community are also interested in eye tracking data. Saliency models predicting fixation distribution and saccadic models predicting scanpaths are two important topics in computer vision. While fixation density map (21) has been widely accepted as the baseline to evaluate saliency model performance, few efforts are dedicated into finding an appropriate baseline for saccadic models. Generally, researchers obtain the upper bound of scanpath prediction performance based on inter-observer consistency and choose from individual scanpaths the one that is the closest to the rest on behalf of all the scanpaths for visualization (22). Similar to STA, the inter-observer consistency method (IOC) also preprocesses recorded individual scanpaths into sequences based on clustering results. Scanpath similarity is measured by Needleman-Wunsch string matching algorithm. Such simplification retains the viewing order but abandons the spatial distribution of scanpaths.

However, it is fixation order and fixation distribution that jointly determine scanpath shape. So some researchers adopted Dynamic Time Warping (DTW) (23) to directly compare scanpaths without preprocessing or simplication (24). In our previous work (25), we proposed the Candidate-constrained DTW Barycenter Averaging (CDBA) algorithm to take into account spatial distribution when analyzing the viewing trend. But still there is little discussion about the important role that gaze duration plays in characterizing scanpaths. Hence, in this paper we extend the framework to generalize viewing trends in not only scanpath shape but also gaze duration. Experiments are conducted to assess the ability of obtained scanpaths to reflect viewing patterns.

## Methodology

**Figure 2.**
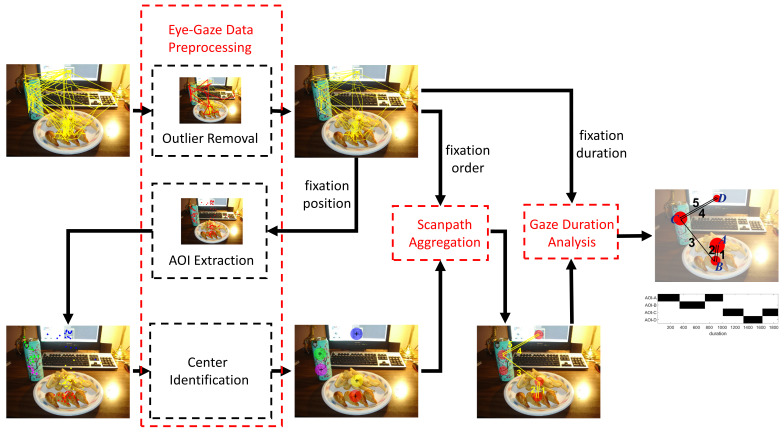
The extended framework to find a representative scanpath that shows attention distribution, attention shift as well as attention span.

The overall framework to obtain the representative scanpath is shown in Figure 2. It consists of three steps: eye-gaze data preprocessing, scanpath aggregation and gaze duration analysis. Fixation position, order and duration are fully exploited to identify the viewing pattern. The preprocessing step is divided into three substeps: outlier removal, AOI extraction and center identification. The second step focuses on scanpath shape, in which multiple scanpaths are aggregated into a single one. Finally, based on the aggregated scanpath, we analyze the pattern from the perspective of gaze duration and combine the analysis results from all three aspects to obtain the representative scanpath.

### Eye-gaze Data Preprocessing

Eye-gaze data are generally expressed by sequences of fixations. Each fixation is recorded as a point with coordinates and gaze duration. The preprocessing step makes preparation for the next pattern mining procedures: outlier removal ensures the consistency of remaining scanpaths, AOI extraction facilitates a higher-level representation, center identification retains the spatial distribution of scanpath components.

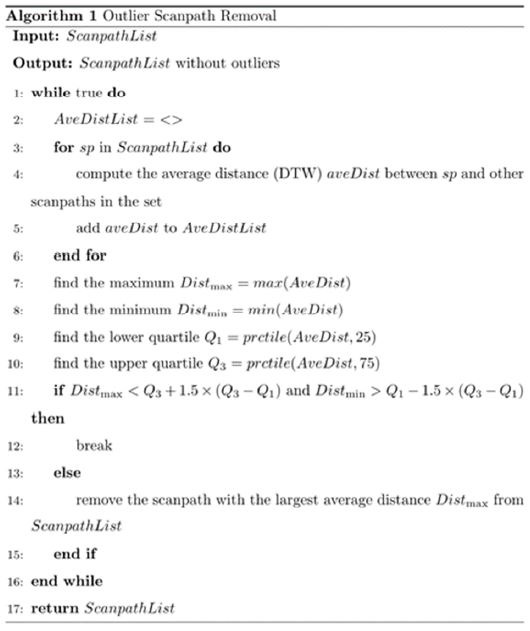



**Outlier Removal**. With different preferences, subjects allocate fixations in irregular and idiosyncratic manners. In addition, inevitable errors in eye tracking and data processing increase the uncertainty of recorded fixations. Therefore, fixations that are isolated might come from interesting viewing behaviors of subjects or measurement errors of eye trackers, leading to discrepancy among scanpaths. Even fixation distributions are similar, how fixations are sequentially arranged to reflect the actual viewing process still varies with different individuals. Therefore, both fixation position and order are potentially causes for scanpath inconsistency.

To eliminate the influence of outlier scanpaths on both spatial distribution and temporal order, we exclude outlier scanpaths with boxplot at the very beginning. Boxplot is a statistical tool that enables us to detect outliers and observe the dispersion degree of data. Algorithm 1 explains how the boxplot works in detail. In Algorithm 1, we use Dynamic Time Warping (DTW) (23) to calculate the distance or dissimilarity

between any two scanpaths. Outlier removal guarantees inter-observer consistency to some degree so the result pattern can reflect the common trend from the compatible majority.

**AOI Extraction**. According to Gestalt theory (26), the nature of unified whole is not simply the addition of its parts. So visual attraction is not from a single pixel but a whole region of interest. It is possible that for the same visual target, fixations scatter on different locations due to the high degree of viewing freedom. As a result, fixation based scanpaths do not facilitate an abstract expression, making it hard to identify what is common in eye tracking data. Therefore, we should express the representative scanpath by higher level components such as AOIs. For example, ScanMatch (27) algorithm uses grid mask to transform fixation based scanpaths to AOI sequences. But the number of grids is flexibly determined and AOIs are not associated with image content. Considering that fixations are stimulus-driven, the clustering structure of fixations is closely related to the distribution of visual attraction. Hence, the representative scanpaths we discuss in this paper are composed of AOIs that are associated with fixation clusters.

All the fixation points are clustered by the algorithm proposed by Rodriguez et al. (28), which considers two properties of points: local density ρ and distance from points with higher density δ.

Fixations with large values of ρ and δ are recognized as cluster examplars. To determine the number of clusters, γ=ρ×δ is calculated for each fixation and all the values are sorted in decreasing order. Then a threshold is set so that fixations with γ larger than the threshold stick out and cluster number is accordingly determined. The threshold can be set as the arithmetic mean or geometric mean empirically. In our experiment, we used the weighted geometric mean, which is calculated as follows:

**(1):**
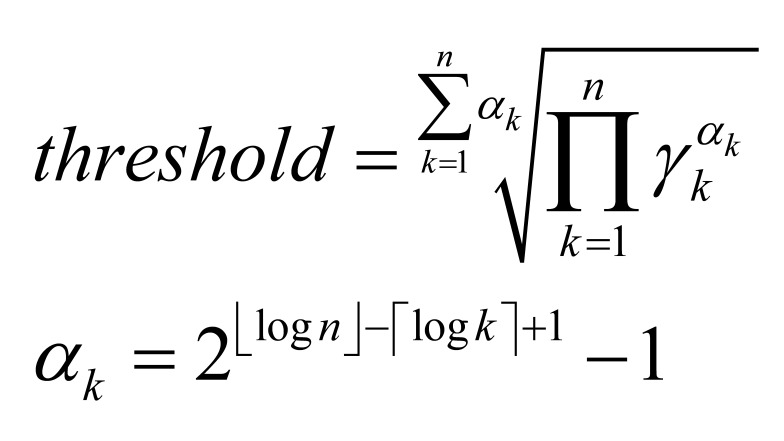


where $\gamma_{1}$, $\gamma_{2}$,…, $\gamma_{n}$ have been sorted in decreasing order. The weighted geometric mean puts more emphasis on larger γ and leads to fewer and less overlapped clusters than the geometric mean.

**Center Identification**. Now all the fixations are assigned to different AOIs. To retain the spatial information of scanpaths, we need to take into account the locations of AOIs. Instead of simply averaging coordinates or choosing points with large γ as centers, we adopt a random walk based method (29) to identify AOI centers, which is more robust and less likely to be affected by edge points of a cluster. The random walk based method aims to obtain a coefficient l for each fixation in the AOI and calculates the weighted center as the final AOI center.

The coefficient l is updated by the following formula:

**(2):**
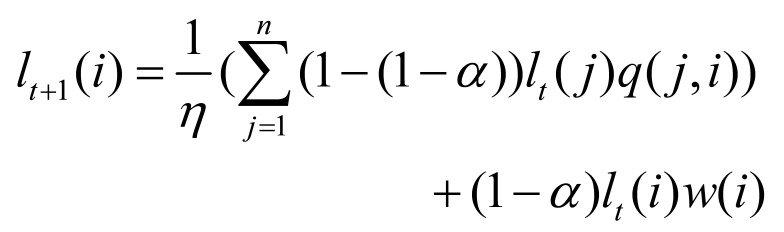


where w(i) is the initial coefficient of fixation i defined by fixation density, η is the normalizing parameter, q(j,i) is the transition probability from fixation i to fixation *j*.

**(3):**
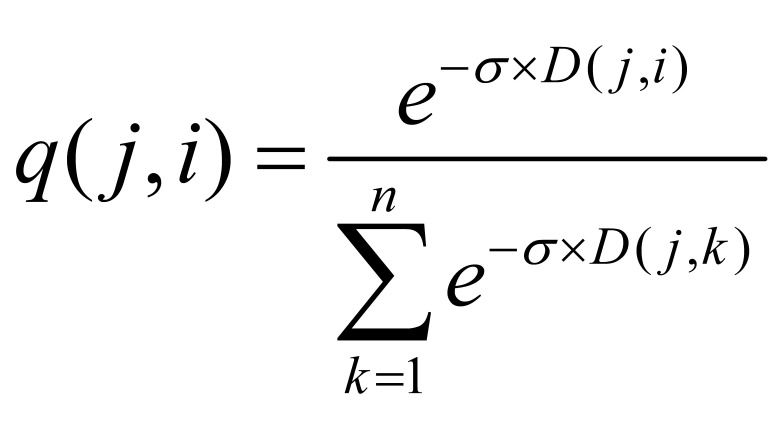


where D(j,i) is the Euclidean distance from fixation j to fixation i, σ is introduced to influence the center distribution subtly.

Different from simple segmentation or grid mask that only allows scanpaths to be treated as character strings, AOI centers make it possible to denote scanpaths by sequences of coordinates and thus can also be regarded as indicators of AOI distribution. AOIs with identified centers are considered as candidate components for the representative scanpath in the aggregation stage.

### Scanpath Aggregation

Generally speaking, the barycenter of points in a cluster is regarded as the representative or examplar of the cluster. Likewise, we aggregate multiple scanpaths into a single one by computing the “barycenter” of the scanpath set. In other words, we try to calculate a representative scanpath that is the closest to individual scanpaths in terms of average distance. Mathematically, the representative scanpath is defined as follows:

**(4):**
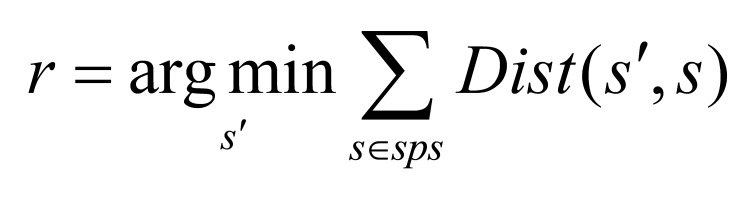


where r is the representative scanpath, $s^{\prime }$ is any scanpath that may become the representative scanpath, s is an individual scanpath in the given scanpath set sps, and Dist is a function calculating the distance or dissimilarity between two scanpaths.

Here we utilize Dynamic Time Warping (DTW) to measure scanpath distance. DTW was first put forward for speech recognition and then widely used in time series analysis (30). Traditional string matching algorithms like Needleman-Wunsch algorithm (31) and Levenshtein Distance (32) simply treat scanpaths as strings and need to additionally construct a cost matrix to take into account spatial proximity, while DTW already involves the construction. In most cases, scanpaths are recorded as sequences of components with coordinates. Given two scanpaths $A=A_{m}=<a_{1},\;a_{2},\cdots ,a_{m}>$ and $B=B_{n}=<b_{1},\;b_{2},\cdots ,b_{n}>$, the DTW distance is recursively computed by the following formula:

**(5):**
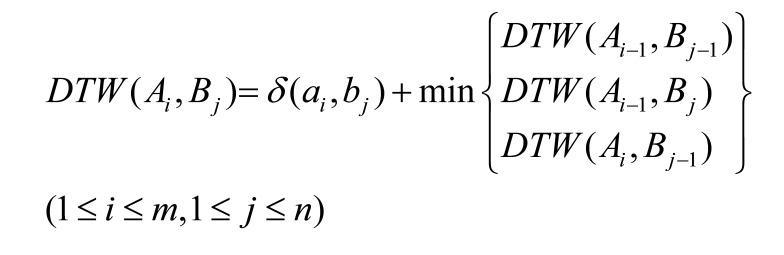


where $A_{i}$, $B_{j}$ are the subsequences of A and B, $a_{i}$ and $b_{j}$ are components of scanpaths A and B respectively, δ() is the Euclidean distance function. The distance or dissimilarity between scanpath A and B is:

**(6):**
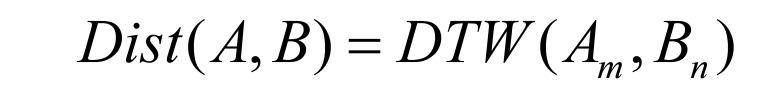


It is difficult to directly get the optimal solution of Equation (1). Hence, we add the following constraints to make it feasible:

- The representative scanpath must be composed of abstract scanpath components such as AOIs;
- Any two contiguous components in the representative scanpath must be contiguous in at least one individual scanpath;
- The occurrence count of each component in the representative scanpath does not exceed the maximum occurrence count of the component in all the individual scanpaths.

These constraints not only simplify the aggregation but also force the obtained scanpath to be more reasonable. The first constraint guarantees the aggregated scanpath is expressed at a higher level. The second and the third constraints ensure that the aggregated scanpath will not deviate too far from individual scanpaths. We propose two methods for scanpath aggregation.

**Heuristic Method**. The heuristic method first constructs a candidate set for each AOI. The candidate set contains all the potential subsequent AOIs for a certain AOI. In other words, AOIs in the candidate set for ${\text{AOI}}_{i}$ must follow ${\text{AOI}}_{i}$ in at least one individual scanpath. Then all the possible scanpaths are enumerated by extending scanpaths of 1 fixation to scanpaths of n fixations. A scanpath is extended by choosing an AOI from the candidate set of the last AOI on the scanpath and adding it to the end. When the occurrence count of a certain AOI is equal to its maximum occurrence count in individual scanpths, the AOI is removed from the candidate set and thus will not appear in later enumerated scanpaths. Finally, the scanpath with the smallest DTW from individual scanpaths is chosen from all the enumerated scanpaths as the representative. *n* is the specified maximum fixation number. When *n* is large enough, we can get the theoretically optimal result for Equation (2), which provides a lower bound of the average distance.

**Candidate-constrained DTW Barycenter Averaging (CDBA) algorithm**. Since the heuristic method is time and space consuming, we propose another algorithm for scanpath aggregation by imposing some constraints on the DTW Barycenter Averaging (DBA) algorithm (33) as an approximation (25). CDBA also needs to construct a candidate set for each AOI and adjust the set members like the heuristic method. Then it defines an initial average scanpath as the reference scanpath and then updates the reference scanpath iteratively. For each iteration, CDBA consists of two steps: computing DTW between every individual scanpath and the reference scanpath and updating the components of the reference scanpath.

- DTW computation. When computing DTW between two sequences, we can obtain the accumulation matrix and find the path of cost accumulation, which indicates the optimal alignment between sequences. The process of DTW computation is repeated between every actual scanpath and the reference scanpath.
- Scanpath update. In the update step, each component of the reference scanpath is updated by the “constrained barycenter” of fixations that are aligned to it during the computation process. The “constrained barycenter” means an AOI belonging to the candidate set and having the minimum average distance with all the aligned fixations.

The above two steps are repeated until the reference scanpath does not change. The process of CDBA is summarized in Algorithm 2.

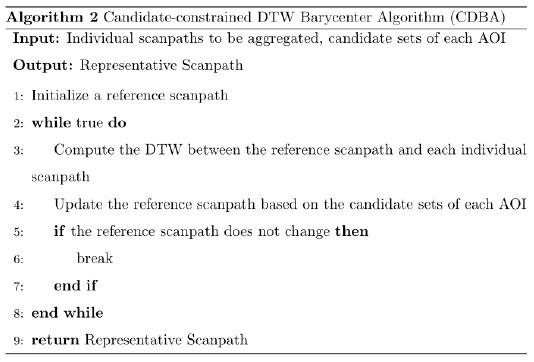



### Gaze Duration Analysis

After scanpath aggregation, we obtain an aggregated scanpath that can tell us not only which areas draw our attention but also the priority of attraction. In this section, we aim to embed gaze duration into the aggregated scanpath. To specify how long an AOI can hold our attention, we transform each individual scanpath (of fixations) into an AOI sequence (of clusters) and statistically analyze the gaze duration of each AOI for all the individual scanpaths. The gaze duration of each AOI in the aggregated scanpath is obtained by averaging the gaze duration of the same AOI in all the individual sequences. Note that when we analyze AOI duration, one and the same AOI appearing more than once in a sequence is regarded as different AOIs and will be distinguished by their appearing order in the sequence.

## Eye Tracking Study

### Eye Tracking Data

To investigate the rationality of representative scanpaths, we conduct experiments on two large public eye-tracking data sets, namely OSIE data set (34) and MIT1003 data set (35).

- OSIE Data Set contains 700 images. Each image is freely viewed by 15 subjects for 3 seconds. All the images are of the size 800×600 pixels.
- MIT1003 Data Set includes 1003 scenes freely viewed by 15 subjects for 3 seconds. The longest dimension of each image is 1024 pixels.

### Procedure

**Figure 3.**
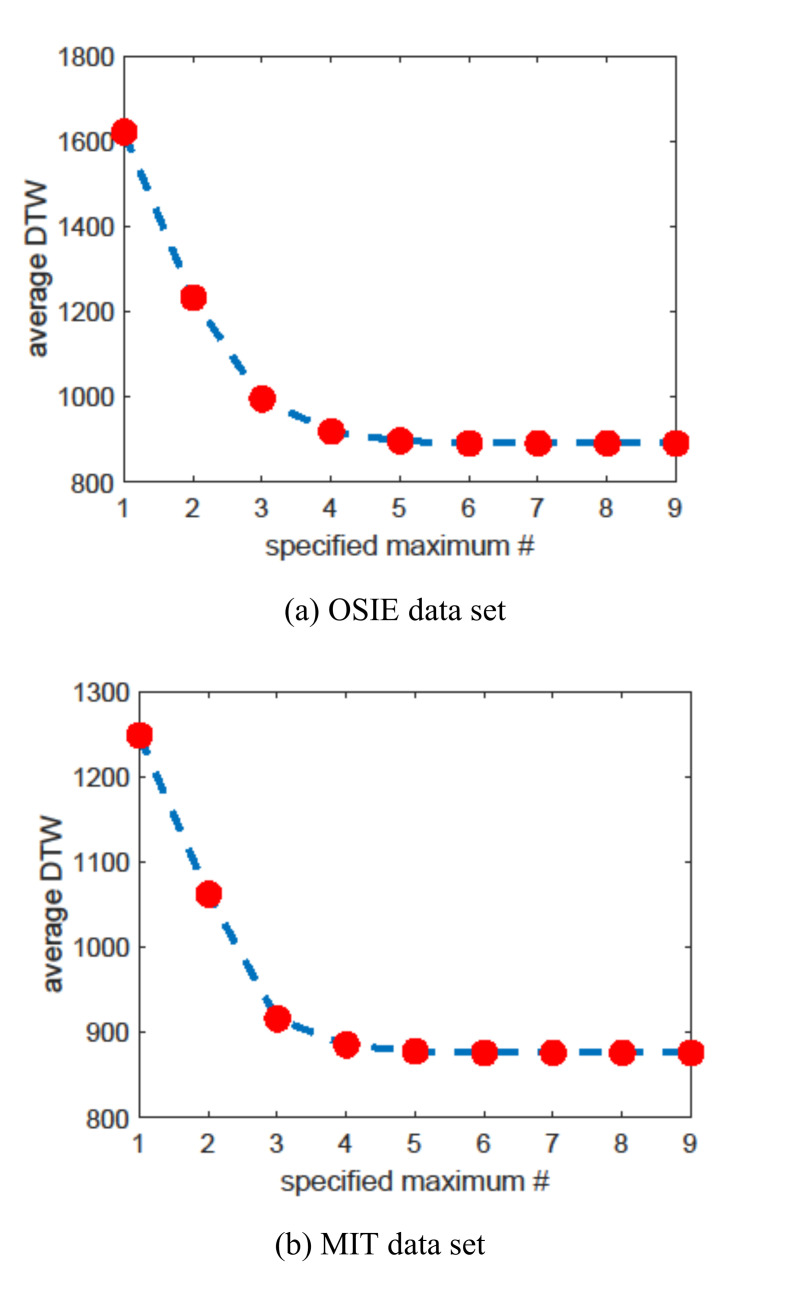
Average DTW varying with the specified maximum fixation number.

The key process in our framework is scanpath aggregation, which can be substituted by other methods like eMine (11), STA (13), SPAM (20) and IOC (22). The first three of them can not directly operate on scanpaths consisting of fixations with coordinates and need to convert scanpaths into character strings. IOC also relies on some preprocessing steps for scanpath quantization. To make sure the comparison is fair, we adopt the same preprocessing step in our framework. The outlier removal process averagely excludes 0.61 and 0.86 scanpaths per image for OSIE and MIT 1003 data sets, respectively. In addition, despite the outlier removal process, eMine still fails to produce any result for some images, so for eMine algorithm, we only consider cases in which eMine algorithm has final outputs. For SPAM algorithm, we set the minimum supporting number of subjects as the half of the total number, which may lead to more than one frequent subsequences, so we choose from these frequent subsequences the one that is optimal with regard to Equation (1) as the representative scanpath. For IOC algorithm, we adapt it for our framework by taking DTW as its distance function and choosing the scanpath with the smallest average DTW. For the heuristic method, we need to determine the specified maximum number n when enumerating all the possible scanpaths. Figure 3 shows average DTW varying with given maximum length n. For both data sets, when n is equal to or larger than 8, the average DTW does not change and the heuristic method can get the theoretically best results. So the maximum number is set as 8 in later discussion for the heuristic method unless otherwise stated.

Due to the high degree of viewing freedom, it is hard to define ground truth representative scanpaths. The only way to evaluate the rationality of the obtained scanpath is to compare it against each individual scanpath with the standard string-edit algorithm as suggested by Eraslan et al. (13, 14). More sophisticated methods to compare scanpaths like ScanMatch (27), MultiMatch (36), and ScanGraph (37) are also developed, facilitating the evaluation.

In our experiment, the evaluation of representative scanpaths is conducted at three different levels:

- Scanpath length: scanpath length reflects the frequency of attention shift, so we compare the length distribution to check whether representative scanpaths can reflect this property;
- Scanpath shape: scanpath shape, partly influenced by scanpath length, is related to both spatial distribution and temporal order, which is measured by DTW in our experiment;
- Overall scanpath similarity: overall scanpath similarity comprehensively considers scanpath shape and gaze duration. ScanMatch and MultiMatch can provide such comparison.

### Results

**Figure 4.**
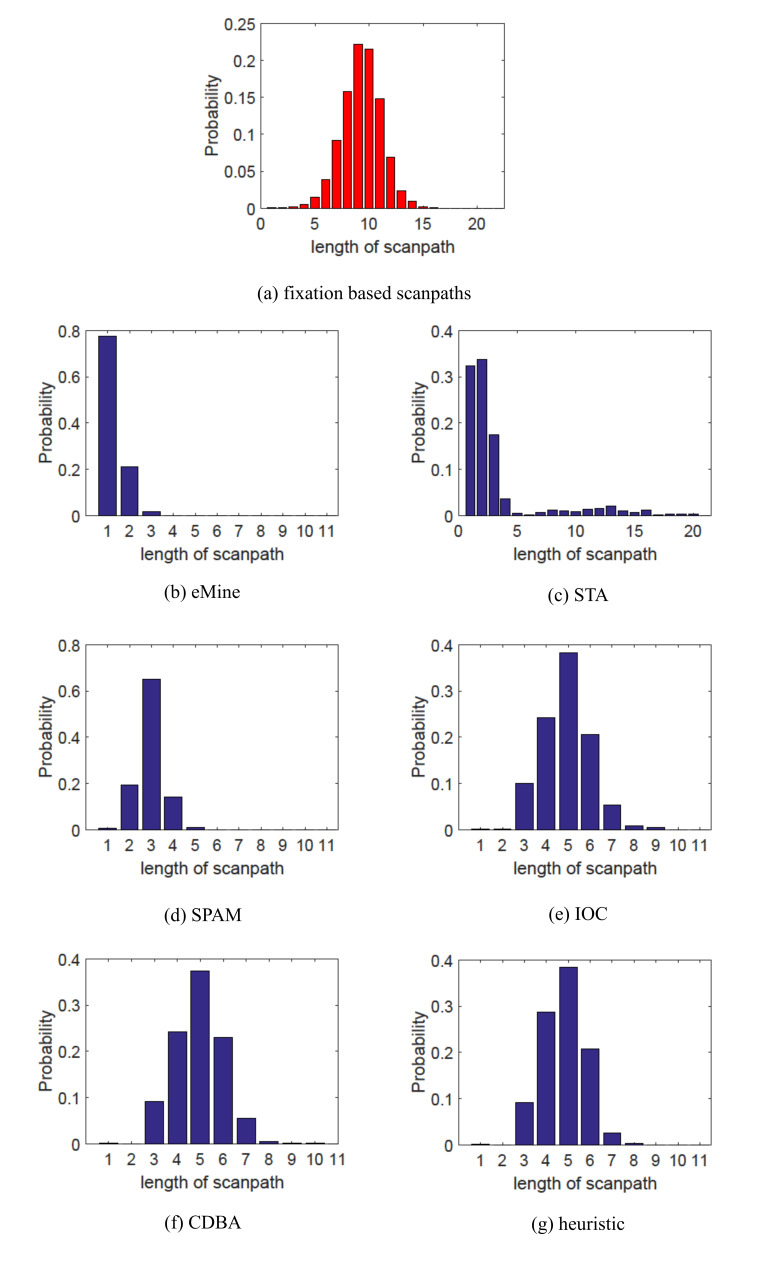
Length distribution of individual scanpaths and aggregated scanpaths for OSIE data set.

**Figure 5.**
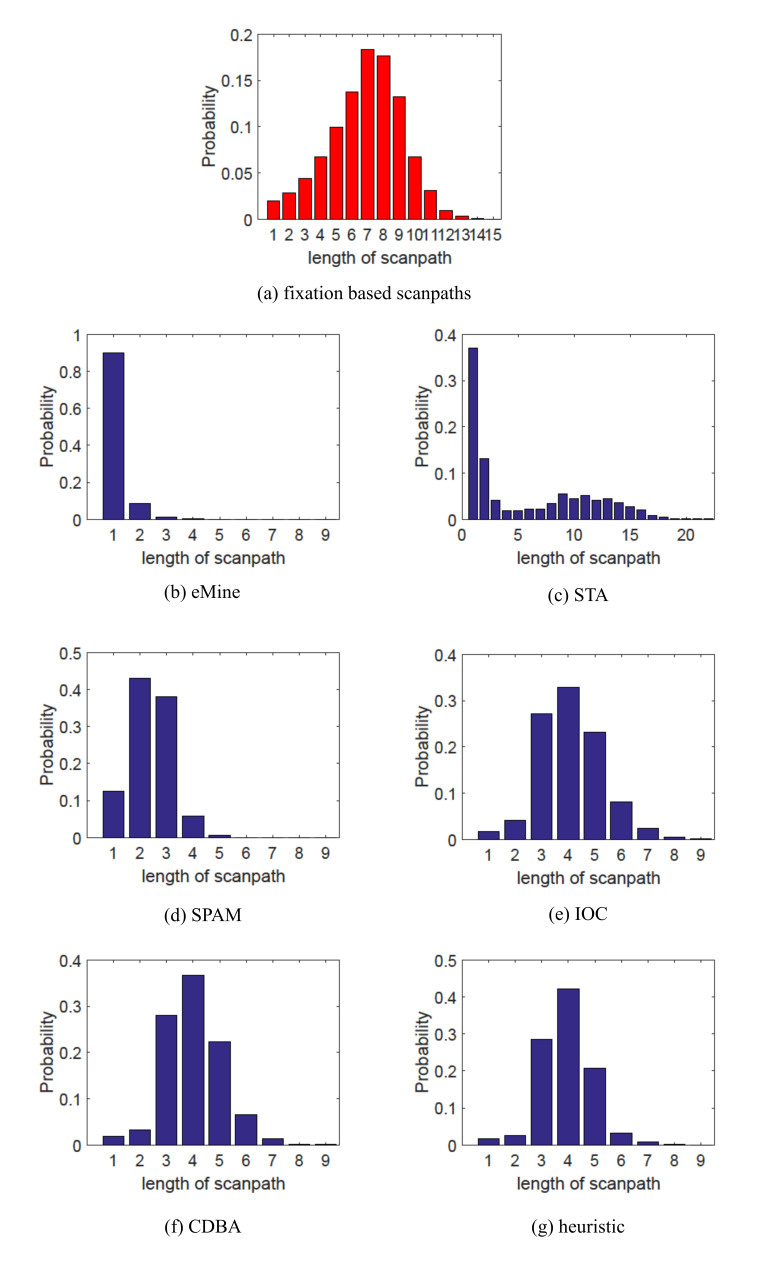
Length distribution of individual scanpaths and aggregated scanpaths for MIT1003 dataset.

**Analysis of Scanpath Length**. Scanpath length reflects the frequency of attention shift. Figure 4 and Figure 5 analyze the length of representative scanpaths for both OSIE and MIT1003 datasets. From Figure 4 (a) and Figure 5 (a), we can find that length distributions of individual scanpaths are similar to normal distribution, which indicates that for only a small number of images, people concentrate on certain areas (hardly shift) or roam over the whole image (frequently shift) while for most images the shift frequency is relatively stable, neither too large nor too small. Thus the bell-shaped property should also be reflected by representative scanpaths. Considering that all the representative scanpaths are AOI based while individual scanpaths are fixations based, the absolute values of scanpath length may be different but the bell-shaped property of scanpath length distribution should be kept. However, eMine, STA and SPAM fail to retain this property and obtain right-tailed distributions. All of them are more likely to get shorter representative scanpaths, which reflect the pattern that for most images, subjects tend to concentrate on certain areas and hardly shift their attention. IOC, CDBA and the heuristic method can keep the bell-shaped distributions.

**Analysis of Scanpath Shape**. In this part, we evaluate the ability of representative scanpaths to reflect attention distribution and attention shift, that is, the shape of representative scanpaths. We measure this ability by computing the average distance (DTW) between the representative scanpath and all the actually recorded scanpaths as suggested by Le Meur et al. (24). Quantitative results are shown in Table 1. A smaller DTW means a better result. The average DTW between representative scanpaths obtained by the heuristic method and all the recorded scanpaths is the smallest. In other words, the heuristic method produces the best solutions for Equation (1), followed by CDBA and IOC. The results of statistical analysis are presented in Table 2, which shows there is a significant difference between the results of our proposed “barycenter” based methods (CDBA and heuristic) and other methods.

**Analysis of Overall Scanpath Similarity**. In this part, we estimate and assign gaze duration to scanpaths obtained in the aggregation step. Note that none of the existing algorithms except for STA have discussed representative scanpaths with gaze duration. Even though STA employs the duration information when identifying trending elements, it still focuses on the analysis of trending scanpaths and does not further analyze gaze duration. To make fair comparisons, we combine our gaze duration analysis method with all the methods proposed for scanpath aggregation, i.e., eMine, SPAM, STA and IOC. The overall scanpath similarity is evaluated by MultiMatch (Jarodzka et al., 2010) and ScanMatch (Cristino et al., 2010). MultiMatch compares scanpaths from five aspects: vector similarity, direction similarity, length similarity, position similarity and duration similarity. ScanMatch only outputs an integrated score reflecting order consistency, spatial proximity and duration similarity. The parameters involved in ScanMatch implementation are set as follows: Xbin = 24, Ybin = 18, Threshold = 3.5, GapValue = 0, TempBin = 100 (TempBin =0 when duration is not taken into account). We compare the representative scanpath with each actually recorded scanpath using both algorithms and compute the average scores. Table 3 shows the results on both datasets. The larger the scores, the better the results. Our proposed methods (CDBA* and Heuristic*) still outperform eMine, STA and SPAM, but the advantages of our methods over IOC are not so obvious. Then we further conduct statistical test on the ScanMatch results (with duration). The difference between the proposed methods and the first three methods, i.e., eMine, STA and SPAM, is significant on both data sets but this is not the case with IOC. It can be seen that although the heuristic method can get a smaller average distance in terms of DTW, scores of MultiMatch and ScanMatch are neck and neck with CDBA and IOC on both datasets. This may be caused by the fact that DTW directly takes Euclidean distance as elements in the cost matrix while both MultiMatch and ScanMatch conduct scanpath simplification or quantization before comparison.

**Table 4.** Average DTW on two data sets ↓

Dataset	eMine	STA	SPAM	IOC	CDBA	heuristic
OSIE	1644	1418	1050	921	899	891
MIT1003	1319	1467	1007	910	882	876

**Table 2.** The Statistical Test Results of DTW. NA: Not applicable because df is not related to the Wilcoxon Test. N: the number of images for which both comparison algorithms can find the representative scanpaths.; ***: p<0.0001.

Dataset	Algrotihm	Test	N	df	T or Z value	Effect Size
OSIE	CDBA-eMine	Wilcoxon	531	NA	-19.9092***	-1.3536
	CDBA-STA	Wilcoxon	700	NA	-22.8043***	-1.1528
	CDBA-SPAM	Wilcoxon	700	NA	-21.7021***	-0.5653
	CDBA-IOC	Wilcoxon	700	NA	-14.4308***	-0.1025
	Heuristic-eMine	Wilcoxon	531	NA	-19.3612***	-1.3612
	Heuristic-SPAM	Wilcoxon	700	NA	-21.8317***	-0.5999
	Heuristic-STA	Wilcoxon	700	NA	-22.8062***	-1.1689
	Heuristic-IOC	Wilcoxon	700	NA	-18.2585***	-0.1443
	Heuristic-CDBA	Wilcoxon	700	NA	-13.6244***	-0.0420
MIT1003	CDBA-eMine	Wilcoxon	484	NA	-18.6447***	-0.9658
	CDBA-STA	Wilcoxon	1003	NA	-27.0153***	-1.0299
	CDBA-SPAM	Wilcoxon	1003	NA	-25.1192***	-0.4019
	CDBA-IOC	Wilcoxon	1003	NA	-19.7691***	-0.1033
	Heuristic-eMine	Wilcoxon	484	NA	-18.6447***	-0.9761
	Heuristic-STA	Wilcoxon	1003	NA	-27.1454***	-1.0398
	Heuristic-SPAM	Wilcoxon	1003	NA	-25.3895***	-0.4236
	Heuristic-IOC	Wilcoxon	1003	NA	-22.3411***	-0.1273
	Heuristic-CDBA	Wilcoxon	1003	NA	-15.3338***	-0.0243

**Table 3.** Evaluating the representative scanpath by MultiMatch and ScanMatch ↑ (* means the aggregation algorithm combined with the proposed duration analysis method)

Dataset	Algorithm		MultiMatch				ScanMatch	
		vector	direction	length	position	duration	without duration	with duration
OSIE	eMine*	0.181	0.123	0.191	0.176	0.130	0.120	0.219
	STA*	0.567	0.402	0.604	0.550	0.409	0.199	0.311
	SPAM*	0.853	0.655	0.891	0.837	0.602	0.244	0.386
	IOC*	0.881	0.744	**0.906**	0.871	0.613	0.348	0.474
	CDBA*	**0.882**	**0.749**	0.905	**0.875**	**0.614**	**0.351**	**0.476**
	Heuristic*	**0.882**	**0.749**	0.905	0.874	**0.614**	0.344	0.474
MIT1003	eMine*	0.083	0.058	0.088	0.080	0.060	0.149	0.224
	STA*	0.524	0.399	0.542	0.503	0.398	0.251	0.275
	SPAM*	0.734	0.539	0.754	0.711	0.555	0.254	0.324
	IOC*	0.842	0.696	0.849	0.819	0.620	**0.355**	**0.419**
	CDBA*	**0.843**	0.701	0.849	0.820	0.617	0.352	0.416
	Heuristic*	**0.843**	**0.702**	**0.850**	**0.821**	**0.620**	0.349	0.415

**Table 4.** The statistical test results of ScanMatch scores (with duration). NA: Not applicable because df is not related to the Wilcoxon Test. N: the number of images for which both comparison algorithms can find the representative scanpaths. *: p<0.05; ***: p<0.0001.

Dataset	Algrotihm	Test	N	df	T or Z value	Effect Size
OSIE	CDBA-eMine	Paired t-test	531	530	54.1696***	1.6125
	CDBA-STA	Wilcoxon	700	NA	22.6372***	1.2585
	CDBA-SPAM	Wilcoxon	700	NA	20.9515***	0.8052
	CDBA-IOC	Wilcoxon	700	NA	1.7021	0.0279
	Heuristic-eMine	Wilcoxon	531	NA	19.8712***	1.6068
	Heuristic-STA	Wilcoxon	700	NA	22.5733***	1.2461
	Heuristic-SPAM	Wilcoxon	700	NA	20.9413***	0.7875
	Heuristic-IOC	Wilcoxon	700	NA	0.1103	0.0057
	Heuristic-CDBA	Wilcoxon	700	NA	-1.8394	-0.2222
MIT1003	CDBA-eMine	Wilcoxon	484	NA	18.6006***	1.3676
	CDBA-STA	Wilcoxon	1003	NA	26.6705***	1.0802
	CDBA-SPAM	Wilcoxon	1003	NA	24.0124***	0.7038
	CDBA-IOC	Wilcoxon	1003	NA	-2.0228*	-0.0253
	Heuristic-eMine	Wilcoxon	484	NA	18.6003***	1.3736
	Heuristic-STA	Wilcoxon	1003	NA	26.5807***	1.0766
	Heuristic-SPAM	Wilcoxon	1003	NA	23.9828***	0.6980
	Heuristic-IOC	Wilcoxon	1003	NA	-2.4115*	-0.0353
	Heuristic-CDBA	Wilcoxon	1003	NA	-1.1309	-0.0099

### Summary

In our experiment, we can regard the adaptation of IOC as constructing a candidate set that contains AOI-level scanpaths transformed from individual fixation-level scanpaths. In other words, IOC actually finds an optimal solution of Equation (1) under stricter constraints. In addition, CDBA and the heuristic method are also based on Equation (1), and the outputs of CDBA can actually be regarded as approximations of the heuristic results. Compared with the heuristic method, IOC chooses from a smaller candidate set while CDBA searches the set in a more efficient way, but these three algorithms share a similar idea, choosing a scanpath from a candidate scanpath set as the representative. In this sense, all the algorithms we discussed above can be categorized as follows: (1) “barycenter” based: IOC, CDBA, heuristic; (2) subsequence based: eMine, SPAM; (3) others: STA.

When evaluated by scanpath length, the “barycenter” based method can well keep the bell shaped distribution of scanpath length. The comparison by DTW also indicates that all the “barycenter” based methods can produce representative scanpaths similar to actually recorded individual scanpaths in scanpath shape. As for overall scanpath similarity, the “barycenter” based methods improve the performance by a large margin over others, which consolidates that “barycenter” based aggregated scanpaths are more suitable to be combined with gaze duration to get final representative scanpaths. In summary, representative scanpaths obtained by “barycenter” based methods can better describe viewing patterns.

### Interpretation of Representative Scanpaths

**Figure 6.**
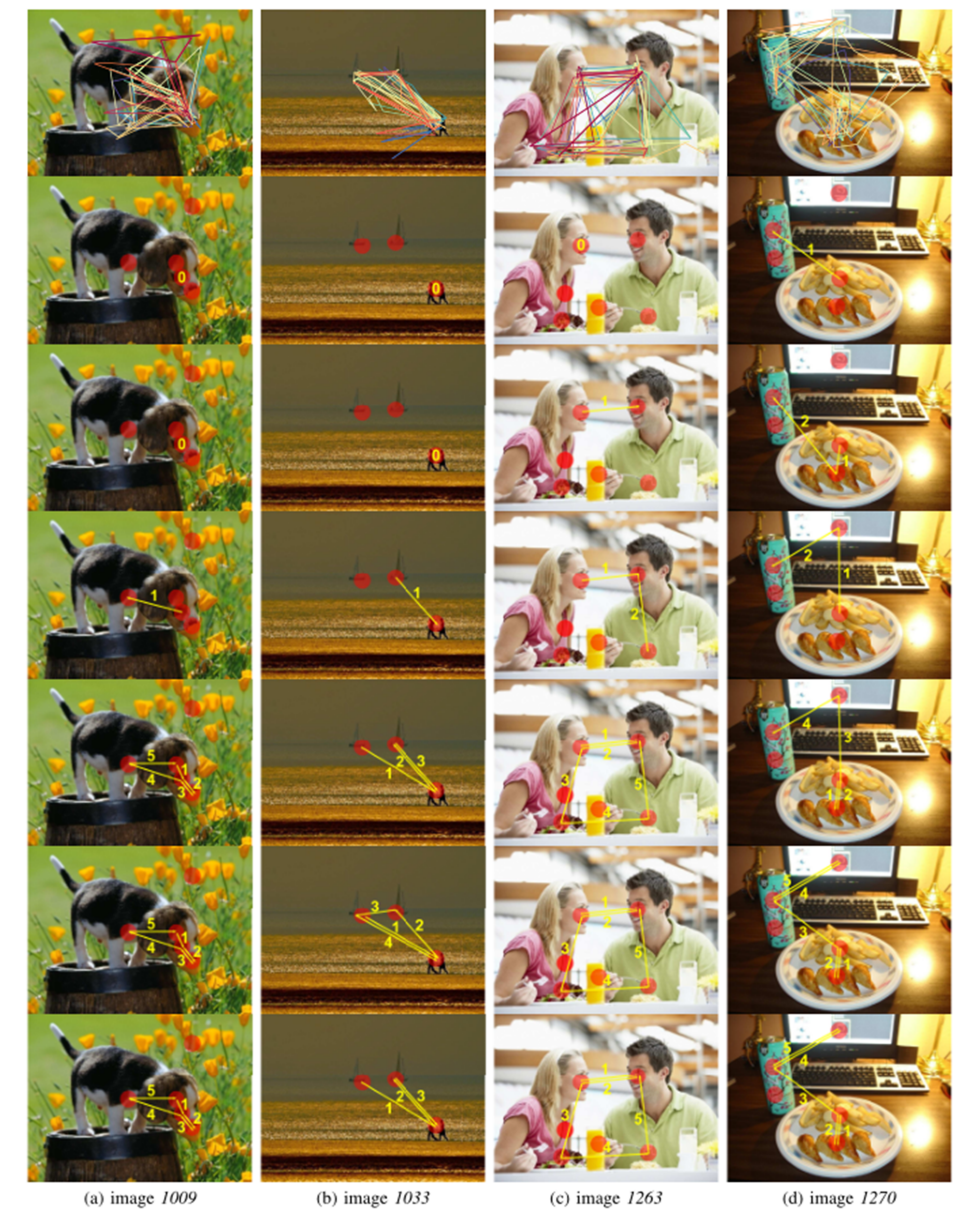
Aggregated scanpaths for four different images from OSIE data set. From top to bottom: individual scanpaths, eMine, STA, SPAM, IOC, CDBA, heuristic.

Figures 6 shows the aggregated scanpaths obtained by different algorithms. In Figure 6, red circles represent AOIs. Yellow arrows indicate the direction and numbers indicate the order. Images 1009 and 1033 respectively contain only one conspicuous foreground object and three objects without many distractors in the background while image 1263 and image 1270 both contain multiple objects with complex background. eMine, STA and SPAM obviously produce shorter scanpaths that may not be able reflect complete viewing patterns. In particular, eMine only identifies one common AOI in all the individual scanpaths and fails to provide any information about attention shift for images 1009, 1033 and 1263. The “barycenter” based methods (IOC, CDBA and the heuristic method) produce identical results for images 1009 and 1263. For image 1009, the representative scanpaths show that attention is first attracted by the dog head, then transferred to the body and finally go back to the head. For image 1263, the pattern is that subjects are first attracted by faces, then linger between faces, next explore objects with which the female and the male are interacting (the food they are eating), and finally redirect their attention to human faces. For images 1033 and 1270, representative scanpaths obtained by IOC, CDBA and the heuristic method are a little different. It is difficult to conclude which scanpath can better describe the viewing pattern since they actually contain some common segments. Take image 1270 for example, all the three representative scanpaths start by an AOI located near image center, which is consistent with the well-known center bias. The main difference between obtained patterns lies in the priority of the AOI on the zip-top can and the AOI on the computer screen. The heuristic method and CDBA prioritizes the AOI on the zip-top can while IOC is on the contrary. Note that there are some letters on the can. Considering text is a top-down factor capable of guiding visual attention (38), the pattern obtained by the heuristic method and the CDBA algorithm may be more reasonable. In addition, although we do not have any so-called ground truth viewing pattern, the identified patterns seem to be congruent with human intuition and some verified findings such as center bias, top-down effect, etc, whether there are one or several foreground objects, simple or complex backgrounds. However, in some cases where the priorities of different visual stimuli are not clear (e.g., image 1033), the identified patterns can only provide limitedly useful knowledge.

**Figure 7.**
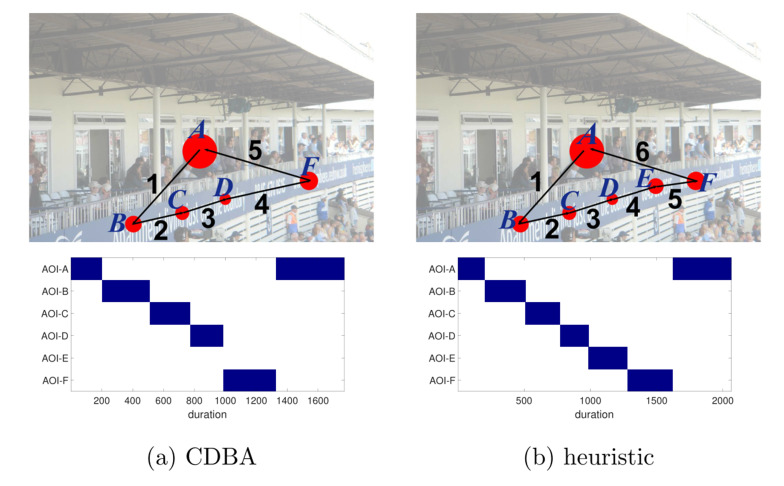
Representative scanpaths with gaze duration of image i1182314083 from MIT data set.

**Figure 8.**
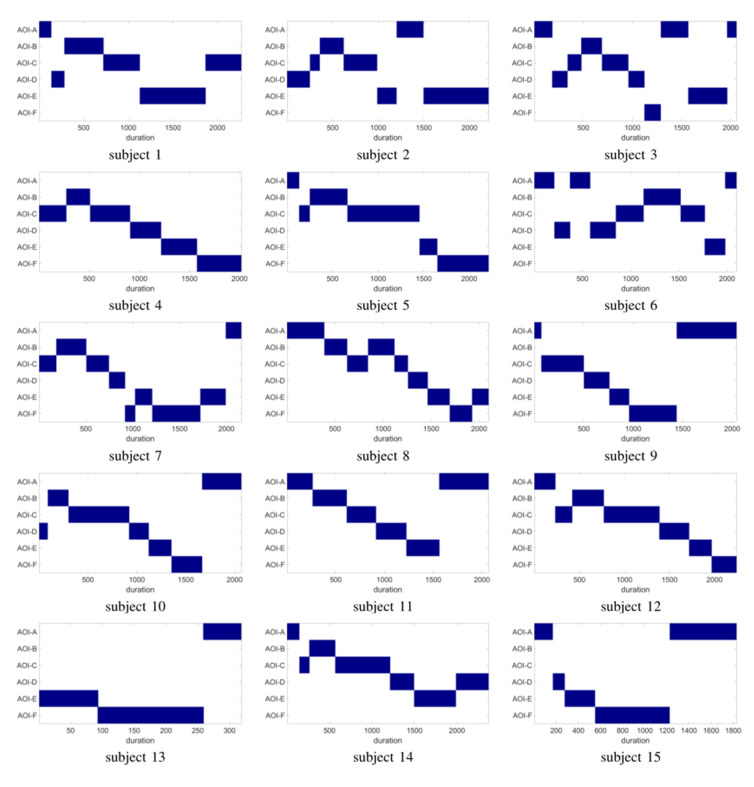
Gaze duration of individual scanpaths of image i1182314083 from MIT data set.

Figure 7 visualizes obtained representative scanpaths obtained by our proposed methods (CDBA and huristic) with duration pattern for image i1182314083 from MIT1003 data set (35). The radius of red circles is proportional to the total gaze duration on the corresponding AOI. Figure 8 shows the duration patterns of individual scanpaths. It is can be seen that the duration pattern of the representative scanpath is visually consistent with the duration pattern of individual scanpaths and can reflect the group trend from an overall perspective.

## Discussion

In this article, we extend our previous framework to identify representative scanpaths from multiple individual scanpaths for natural images. Different from most existing work, we also analyze the duration pattern. The proposed framework consists of three steps: eye-gaze data preprocessing, scanpath aggregation and gaze duration analysis. Experiments demonstrate that our proposed framework is able to identify representative scanpaths reflecting group viewing patterns on natural images.

Based on the algorithms for scanpath aggregation, we further categorize representative scanpaths as follows: (1) “barycenter” based; (2) subsequence based; (3) others. Some algorithms are specially designed to identify viewing patterns on a specific kind of visual stimuli so their performances are not so satisfactory when visual stimuli are changed. For natural images, we find that “barycenter” based representative scanpaths are the closest to individual scanpaths. Such representative scanpaths for natural images are useful in various fields. For example, computer vision researchers attempt to build plausible saccadic models to predict human scanpaths and they need a reliable ground truth scanpath against which predicted scanpaths can be compared. In addition, it is much easier for us to visualize and analyze one representative scanpath than multiple individual scanpaths that are largely overlapped, which makes it possible to validate some assumptions about visual attention and eye movements such as center-bias and top-down bias. The representative scanpath with duration pattern can also give us a hint about what first grabs visual attention and what holds attention for a long period, providing knowledge about what kinds of images are obvious visual attractors.

However, there are some limitations of our work. For example, the eye tracking data set only involves 15 participants, which means there are at most 15 scanpaths for each image. So it is necessary to construct a much larger data set with more participants. The size of the data set can arouse some challenges for the proposed algorithm, like how to efficiently determine the initial reference scanpath for CDBA and how to reduce the space and time cost of the heuristic method. In addition, we use a data-driven approach to obtain AOIs but it could be better to associate AOIs with semantically meaningful objects. The incorporation of semantic segmentation in the preprocessing step needs further investigation.

## Conclusions

Eye tracking data provide insights into how humans perceive and explore their surroundings. Traditional methods to analyze scanpaths target a specific kind of viewing stimuli such as web pages and neglect the duration pattern, so the scanpaths obtained by such methods are not able to reflect the viewing pattern on natural images correctly or comprehensively. In this paper, we extend our previous framework to identify representative scanpaths, considering temporal order, spatial distribution and gaze duration. The framework consists of three steps: eye-gaze data preprocessing, scanpath aggregation and gaze duration analysis. The second step is the key to representative scanpaths identification and can be replaced by traditional methods such as eMine. Based on the algorithms chosen, we further categorize the obtained representative scanpaths as subsequence based, “barycenter” based and others. Experiments demonstrate that our framework can well serve the purpose of generalizing viewing patterns and the “barycenter” based representative scanpaths can better describe the patterns.

## Ethics and Conflict of Interest

The author(s) declare(s) that the contents of the article are in agreement with the ethics described in http://biblio.unibe.ch/portale/elibrary/BOP/jemr/ethics.html and that there is no conflict of interest regarding the publication of this paper.

## Acknowledgements

This work is supported in part by National Natural Science Foundation of China under Grant 61771348 and 61471273, and Wuhan Morning Light Plan of Youth Science and Technology under Grant 2017050304010302.

## References

[b30] Berndt DJ, Clifford J (1994). Using dynamic time warping to find patterns in time series. In: ACM SIGKDD Conference on Knowledge Discovery and Data Mining Workshop.

[b9] Burmester M, Mast M. Repeated Web Page Visits and the Scanpath Theory: A Recurrent Pattern De-tection Approach. Journal of Eye Movement Re-search. 2010; 3(4):5, 1-20. http://dx.doi.org/10.16910/jemr.3.4.5

[b29] Chen, X., & Chen, Z. (2017). Exploring visual attention using random walks based eye tracking protocols. Journal of Visual Communication and Image Representation, 45, 147–155. 10.1016/j.jvcir.2017.02.005 1047-3203

[b27] Cristino, F., Mathôt, S., Theeuwes, J., & Gilchrist, I. D. (2010). ScanMatch: A novel method for comparing fixation sequences. Behavior Research Methods, 42(3), 692–700. 10.3758/BRM.42.3.692 1554-351X 20805591

[b37] Dolezalova J, Popelka S. ScanGraph: A Novel Scanpath Comparison Method Using Visualization of Graph Cliques. Journal of Eye Movement Re-search. 2016; 9(4), 5:1-13. http://dx.doi.org/10.16910/jemr.9.4.5

[b21] Engelke, U., Liu, H., Wang, J., Le Callet, P., Heynderickx, I., Zepernick, H. J., & Maeder, A. (2013). Comparative study of fixation density maps. IEEE Transactions on Image Processing, 22(3), 1121–1133. 10.1109/TIP.2012.2227767 1057-7149 23193452

[b12] Eraslan S, Yesilada Y, Harper S. Eye Tracking Scanpath Analysis Techniques on Web Pages: A Survey, Evaluation and Comparison. Journal of Eye Movement Research. 2016; 9(1):2, 1-19. http://dx.doi.org/10.16910/jemr.9.1.2

[b15] Eraslan S, Yesilada Y, Harper S. Less Users More Confidence: How AOIs don’t Affect Scanpath Trend Analysis. Journal of Eye Movement Research. 2017; 10(4):6, 1-18. http://dx.doi.org/10.16910/jemr.10.4.6 PMC7141090 33828666

[b13] Eraslan S, Yesilada Y, Harper S. Scanpath Trend Analysis on Web Pages: Clustering Eye Tracking Scanpaths. ACM Transactions on the Web. 2016a; 10(4), 20:1-20:35. http://doi.acm.org/10.1145/2970818

[b14] Eraslan S, Yesilada Y, Harper S. Trends in eye tracking scanpaths: Segmentation effect? HT 2016: Proceedings of the 27th ACM Conference on Hypertext and Social Media. 2016b. p. 15-25. http://doi.acm.org/10.1145/2914586.2914591

[b11] Eraslan S, Yesilada Y, Harper S. Identifying pat-terns in eyetracking scanpaths in terms of visual elements of web pages. ICWE 2014: Proceedings of the 14th International Conference on Web Engineering.2014. p. 163-180.

[b16] Eraslan S, Yesilada Y, Harper S. (2017b) Engineer-ing Web-based Interactive Systems: Trend Analysis in Eye Tracking Scanpaths with a Tolerance. EICS 2017: Proceedings of the 9th ACM SIGCHI Sympo-sium on Engineering Interactive Computing Sys-tems. 2017.http:// doi.acm.org/10.1145/3102113.3102116

[b17] Goldberg JH, Helfman JI. Scanpath clustering and aggregation. ETRA 2010: Proceedings of the 2010 Symposium on Eye Tracking Research & Applica-tions. 2010. p. 227-234.http://doi.acm.org/10.1145/1743666.1743721 10.1145/1743666.1743721

[b20] Hejmady P, Narayanan NH. Visual attention pat-terns during program debugging with an ide. ETRA 2012: Proceedings of the 2012 Symposium on Eye Tracking Research & Applications. 2012. p. 197-200. http://doi.acm.org/10.1145/2168556.2168592

[b18] Hembrooke H, Feusner M, Gay G. Averaging scan patterns and what they can tell us. ETRA 2006: Pro-ceedings of the 2006 Symposium on Eye Tracking Research & Applications. 2006. p. 41-41. http://doi.acm.org/10.1145/1117309.1117325 10.1145/1117309.1117325

[b36] Jarodzka H, Holmqvist K, Nyström M. A vector-based, multidimensional scanpath similarity meas-ure. ETRA 2010: Proceedings of the 2010 Sympo-sium on Eye-Tracking Research & Applications. 2010. p. 211-218.http://doi.acm.org/10.1145/1743666.1743718

[b22] Jiang, M., Boix, X., Roig, G., Xu, J., Van Gool, L., & Zhao, Q. (2016). Learning to predict sequences of human visual fixations. IEEE Transactions on Neural Networks and Learning Systems, 27(6), 1241–1252. 10.1109/TNNLS.2015.2496306 2162-237X 26761903

[b35] Judd T, Ehinger K, Durand F, Torralba A. Learning to predict where humans look. ICCV 2009: Proceedings of 2009 IEEE 12th International Conference on Computer Vision. 2009.p. 2106-2113.http://dx.doi.org/10.1109/ICCV.2009.5459462

[b1] Just, M. A., & Carpenter, P. A. (1980). A theory of reading: From eye fixations to comprehension. Psychological Review, 87(4), 329–354. 10.1037/0033-295X.87.4.329 0033-295X 7413885

[b26] Kanizsa, G. (1979). Organization in vision: Essays on Gestalt perception. Praeger Publishers.

[b24] Le Meur, O., & Liu, Z. (2015). Saccadic model of eye movements for free-viewing condition. Vision Research, 116(Pt B), 152–164. 10.1016/j.visres.2014.12.026 0042-6989 25724662

[b32] Levenshtein, V. I. (1965). Binary codes capable of correcting deletions, insertions, and reversals. Soviet Physics, Doklady, 10, 707–710.0038-5689

[b25] Li A, Zhang Y, Chen Z. Scanpath mining of eye movement trajectories for visual attention analysis. ICME 2017:Proceedings of 2017 IEEE International Conference on Multimedia and Expo. 2017. p. 535–540. http://dx.doi.org/10.1109/ICME.2017.8019507

[b8] Magnusson, M. S. (2000). Discovering hidden time patterns in behavior: T-patterns and their detection. Behavior Research Methods, Instruments, & Computers, 32(1), 93–110. 10.3758/BF03200792 0743-3808 10758668

[b10] McClung, S. N., & Kang, Z. (2016). Characterization of visual scanning patterns in air traffic control. Computational Intelligence and Neuroscience, 2016, 8343842. 10.1155/2016/8343842 1687-5265 27239190 PMC4838798

[b3] Mishra A, Kanojia D, Bhattacharyya P. (2016). Predicting readers sarcasm understandability by modeling gaze behavior. AAAI 2016: Proceedings of the 30th AAAI Conference on Artificial Intelligence. 2016. p. 3747-3753.

[b5] Mishra A, Kanojia D, Nagar S, Dey K, Bhattacha-ryya P. Scanpath complexity: Modeling reading ef-fort using gaze information. AAAI 2017: Proceed-ings of the 31st AAAI Conference on Artificial In-telligence. 2017. p. 4429-4436.

[b31] Needleman, S. B., & Wunsch, C. D. (1970). A general method applicable to the search for similarities in the amino acid sequence of two proteins. Journal of Molecular Biology, 48(3), 443–453. 10.1016/0022-2836(70)90057-4 0022-2836 5420325

[b6] Noton, D., & Stark, L. (1971). Scanpaths in eye movements during pattern perception. Science, 171(3968), 308–311. 10.1126/science.171.3968.308 0036-8075 5538847

[b33] Petitjean, F., Ketterlin, A., & Gancarski, P. (2011). A global aver-aging method for dynamic time warping with appli-cations to clustering. Pattern Recognition, 44(3), 678–693. 10.1016/j.patcog.2010.09.013 0031-3203

[b38] Ramanishka V, Das A, Zhang J, Saenko K. (2017) Top-down visual saliency guided by captions. CVPR 2017:Proceedings of 2017 IEEE Internation-al Conference on Computer Vision and Pattern Rec-ognition. 2017. http://dx.doi.org/10.1109/CVPR.2017.334

[b2] Razin Y, Feigh K. Learning to predict intent from gaze during robotic hand-eye coordination. AAAI 2017: Proceedings of the 31st AAAI Conference on Artificial Intelligence.2017. p. 4596-4602.

[b28] Rodriguez, A., & Laio, A. (2014). Machine learning. Clustering by fast search and find of density peaks. Science, 344(6191), 1492–1496. 10.1126/science.1242072 0036-8075 24970081

[b23] Sakoe, H., & Chiba, S. (1978). Dynamic programming algorithm optimization for spoken word recognition. IEEE Transactions on Acoustics, Speech, and Signal Processing, 26(1), 43–49. 10.1109/TASSP.1978.1163055 0096-3518

[b19] West JM, Haake. R, Rozanski EP, Karn KS. eyepat-terns: software for identifying patterns and similari-ties across fixation sequences. ETRA 2006: Pro-ceedings of the 2006 Symposium on Eye Tracking Research & Applications. 2006.p. 149-154. http://doi.acm.org/10.1145/1117309.1117360

[b34] Xu, J., Jiang, M., Wang, S., Kankanhalli, M. S., & Zhao, Q. (2014). Predicting human gaze beyond pixels. Journal of Vision (Charlottesville, Va.), 14(1), 1–20. 10.1167/14.1.28 1534-7362 24474825

[b7] Yarbus, A. L. (1967). Eye Movements and Vision. Plenum Press. 10.1007/978-1-4899-5379-7

[b4] Zhou, L., Zhang, Y., Wang, Z., Rao, L., Wang, W., Li, S., . . . Liang, Z. (2016). A Scanpath Analysis of the Risky Decision-Making Process. Journal of Behavioral Decision Making, 29(2-3), 169–182. 10.1002/bdm.1943 0894-3257

